# Influence of Different Restorative Techniques on the Strength of Endodontically Treated Weakened Roots

**DOI:** 10.1155/2012/343712

**Published:** 2012-05-14

**Authors:** Khalid H. Alsamadani, El-Sayed Mohammed Abdaziz, El-Sayed Gad

**Affiliations:** ^1^Restorative Dentistry, Faculty of Dentistry, Taibah University, Al Madinah Al Monawarah, Saudi Arabia; ^2^Conservative Dentistry Department, Faculty of Dentistry, Ajman University, UAE; ^3^Dental Biomaterials, Restorative Dental Science Department, Faculty of Dentistry, Taibah University, Al Madinah Al Monawarah, Saudi Arabia

## Abstract

*Objective*. Comparing effect of different restoration techniques on fracture resistance of compromised roots. *Methods*. Crowns of 100 single-rooted teeth were sectioned and 10 roots were kept as negative control group (Group 1). Remaining roots were instrumented and divided into one and positive control group of 10 samples (Group 2) and 4 experimental groups of 20 samples each. Group 3: roots were obturated with gutta-percha; Group 4: roots were restored with gutta-percha, composite, and glass fiber post; Group 5: roots were obturated with Resilon; Group 6: Roots were restored with Resilon, composite, and glass fiber post. Roots were weakened before obturation in groups 2, 3, and 5 and after obturation in groups 4 and 6. Fracture strengths were measured using Dartec testing machine and fracture load was recorded in kilo-Newton. Statistical analysis was done using ANOVA and Tukeys test. *Results*. The fractures resistance of restored roots was significantly higher in groups 4, 5, and 6 than in Groups 2 and 3. There were no significant differences between groups 1, 4, 5, and 6. *Conclusions*. Restoration of weakened roots with Resilon or bonding an intermediate composite resin to coronal radicular dentin and to glass fiber post increased their fracture resistance.

## 1. Introduction

Endodontically treated roots with wide and flared canals are at a high risk of fracture as the strength of roots is directly related to the thickness of remaining root dentin. [1–6] These roots may be severely weakened as a result of dental caries that extends deeply into roots, previous endodontic treatment with iatrogenic problems, endodontic treatment of immature roots, internal resorption, and removal of previously placed posts [7–9]. The general loss of tooth structure in the nonvital tooth together with the alterations in collagen distribution may simultaneously contribute to the increased susceptibility of endodontically-treated teeth to fracture under loading. A further reduction in microhardness can be induced by the use of irrigating solutions during endodontic treatment [10, 11]. The loss of water and gutta-percha condensation procedures may also contribute to the weakness reported in endodontically-treated teeth [12, 13].

 In recent years the patient's perception changed and led to a greater demand for preservation of even severely damaged teeth which have been extracted earlier but the restoration of these kinds of teeth still presents a challenge to clinicians. There is a general agreement that endodontic treatment failure is more likely due to restoration failure than endodontic treatment itself. However, it is important to follow a treatment plane with a full respect to the endodontic and restorative techniques. So the final restoration following the root canal treatment is of major importance for a successful outcome otherwise improper restorations may even led to tooth extraction. Gutta-percha with an insoluble root canal sealer can be seen as the gold standard of root canal fillings and is offered the status as a time honored standard for endodontic obturation. The ability of these materials to reinforce an endodontically treated root is discussed with some controversy. 

 Two methods for restoration of weakened root canals were suggested which were conventional and intraradicular reinforcement methods. Conventional methods which include the use of posts or pins are not suitable to restore these weakened roots a variety of reasons. Placement of a retentive pin is not possible because of the lack of dentine substance at the coronal portion of the root. Placement of a cast metal post can cause wedging forces at the already thin and weakened portions of the root and concentrate the stresses at the weakened cervical portion of the root canal due to its higher modulus of elasticity in comparing to surrounding radicular dentin [14–17]. The geometry of the flared canal also results in a very wide, tapered, and un-retentive post. In these situations, if a prefabricated post is used, the excess space within the root canal would be taken up by a bulk of luting cement which could impair the fracture resistance of the root [18, 19]. So, the concept that says post was generally placed in an attempt to strengthen the tooth has “passed.” Post does not strengthen the root, but serves solely to improve retention of the core [20–22]. Thus, these traditional methods of restoration are unsatisfactory and often result in fracture of the root and followed by extraction of the teeth [23].

 The development of an alternative technique, the “Reinforcement Technique” could be implemented for the treatment of such weakened roots [22, 24]. Thus, for a flared and wide canals, it is important that the lost dentin is rebuilt with a strong substitute before placing the post [17].

The using of self-cured composite was firstly introduced to achieve this goal but there was difficulty in controlling the curing time. [25, 26] On the other hand, when light-cured composite resin is used the deep layer of the composite cannot be properly cured because the composite has a limited depth of cure [27]. Using of translucent curing post (Luminex system, Dentatus Ltd, USA) had been widely used clinically and solved the problem of composite curing within deep area of root canal [24, 27]. Following removal of the light transmitting plastic post, a prefabricated glass fiber post of similar size will be cemented with dual-cured resin cement. Such combination has a modulus of elasticity close to dentin, which can reduce the incidence of catastrophic root fracture compared to the usual post crown where stresses are highly concentrated at the coronal third of the roots [28–31].

 Recently, improvements in apical and coronal seals and strengthening of endodontically treated teeth have been proposed by establishing monoblocks via bonding of the root filling materials to intraradicular dentine [32]. This is similar to contemporary adhesive strategies used for intracoronal restorations that attempt to eliminate microleakage and strengthen coronal tooth structures by creating similar monoblocks between tooth substrates and restorative materials [33]. Resilon a thermoplastic synthetic polymer based material introduced in 2004 performs similar to gutta-percha and has the same handling characteristics. A tight adhesion between Resilon cone and the resin-based sealer form a “monoblock” and have potential to strengthen the walls against fracture and decrease the microleakage [34]. There are contradictory results about the strengthening effect of Resilon system on endodontically treated roots and there was, up to our knowledge, no study on the effect of resilon system on the strength of weakened and endodontically treated roots.

The aim of this study was to compare the effect of the following restorative treatment planes on fracture resistance of experimentally weakened roots: (1) obturation of the weakened roots either with gutta-percha or resilon system; (2) Reinforcing the coronal portion of weakened roots (apically filled with gutta-percha or Resilon) with glass fiber posts after relining roots with bonded composite resin that light cured with the aid of transilluminating post.

## 2. Materials and Methods

### 2.1. Teeth Selection

Freshly extracted single-rooted human teeth of similar root length were collected for this study. All teeth were examined under ×25 magnification with digital stereomicroscope (Motic Digital Microscope, Micro-Optic Industrial Group Co. LTD., France) to rule out any tooth with preexisting root fractures. The tooth crown was cut at the cemento-enamel junction using a diamond disc (Brasseler Dental Products, Savannah, GA, USA) to create 15 mm roots. Two angle periapical radiographs were taken for all roots to measure the dentin thickness at the coronal third of root canal. One hundred roots of the same coronal dentin thickness (2.5 mm), apical foramen diameter (0.15 mm), and cervical orifice diameter (2.5 mm) were accepted for the study and divided according to restorative treatment plane into 2 control groups and 4 experimental groups (Table 1).

## 3. Samples Preparations

### 3.1. Root Canal Preparation

Samples in negative control group (Group 1) did not receive any root canal preparation. For the other groups, root canal of each sample was instrumented similarly with rotary nickel-titanium (Ni-Ti) profile system (Dentsply Maillefer, Tulsa, OK, USA) using crown down technique and following manufacturer directions up to master apical file size 35 and taper 0.06. Throughout the instrumentation, each canal was irrigated with 10 mL of 1% sodium hypochlorite using a 27-gauge irrigating needle which inserted approximately to reach 2/3 of root canal. A final irrigation was performed with 5 mL of 17% EDTA (P ulp Dent, Watertown, MA, USA.)

### 3.2. Root Weakening

**Figure 1:**
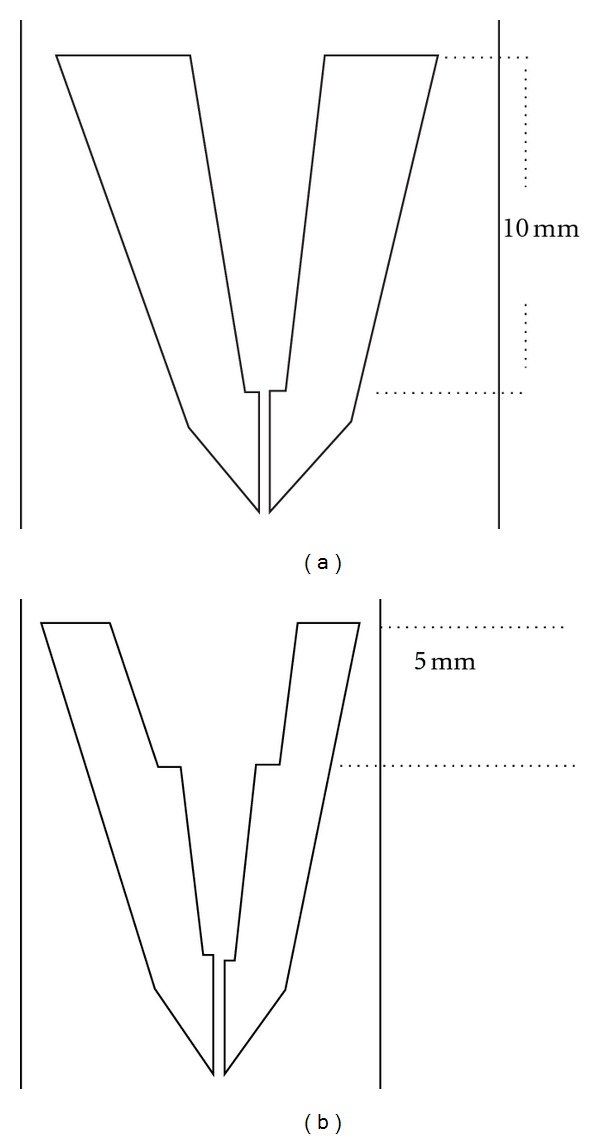
Schematic diagram showing root weakening procedures. (a) Coronal 10 mm of prepared canal was overprepared using luscent helix reamers. (b) A specialized stainless steel bur with diameter 3 mm at the tip and 4.5 mm at body was used to enlarge the coronal 5 mm of previously overprepared canal.

Root weakening was done for groups 2–6 to simulate widely flared and clinically weakened roots. In groups 2, 3, and 5 root weakening was done after finishing root canal preparation and before obturation. For groups 4 and 6 root weakening was done after obturation. The coronal 10 mm of each root either obturated or not obturated was over-prepared (Figure 1(a)) using Luscent Helex Reamer (Dentatus, USA Ltd) in ascending order starting from size 1 (1 mm) till reaching size 6 (1.75 mm) provided that the apical 5 mm of prepared canals was untouched. The coronal 5 mm of widened canals was subjected to further enlargement (Figure 1(b)) using a special low speed diamond bur with a thickness 3 mm at the tip and 4.5 mm at the body, provided that the final thickness of remaining dentin surrounding the canal orifices was 1.5 mm. For Groups 2, 3, and 5, each root canal was recapitulated with the master apical file to remove any packed apical dentin. Then all canals were irrigated with sodium hypochlorite and EDTA solutions and finally rinsed with 10 mL distal water to remove any remaining irrigating solution.

### 3.3. Root Canals Obturation

Regarding to positive control group (Group 2), pre pared weakened roots were kept without obturation. Samples in Groups 3 and 4 were obturated with Gutta-percha and resin sealer while in group 5 and 6 they were obturated with Resilon and Epiphany sealer. Warm vertical compaction technique was used to obturate all samples with the aid of System-B (EIE/Analytic, Orange, CA, USA) and Obtura II system (Obtura Spartan, Fenton, MO, USA).

### 3.4. Root Canal Obturation with Gutta-Percha (Groups 3 and 4)

The resin sealer (Adseal sealer, Meta Biomed co., Ltd, Republic of Korea) was mixed according to manufacturer instructions and placed into the root canal using Lentulo spiral filler (Dentsply Caulk, Milford, DE, USA). The master gutta-percha cone size 35 and 0.06 taper (DiaDent Group International, Republic of Korea) was dipped into the sealer and then inserted within the canal till reaching the working length. A prefitted System-B plugger (SybronEndo, Orange, CA, USA) is activated and then inserted alongside the master cone until reaching 5-6 mm short of working length. The plugger is deactivated at this length and firmly held against the apical gutta-percha for few seconds. Then, the system-B is briefly activated and removed with the coronal part of gutta-percha from the canal. Machtou hand pluggers (Dentsply Maillefer, Baillagues, Switzerland) were used to compact the apical portion of gutta-percha cone until reaching 3 mm short of working length. The remaining coronal portion of the canal space was back filled with warmed gutta-percha released from Obtura II system until reaching coronal orifice.

### 3.5. Root Canal Obturation with Resilon (Groups 5 and 6)

Epiphany self-etch primer (Epiphany, Pentron Dental Product) was applied into the root canals according to manufacturer's instructions. Excess primer was removed with paper point (Dentsply Maillefer, m Tulsa, OK, USA) and the Epiphany sealer was placed with lentulo spiral filler (Dentsply Caulk, Milford, DE, USA). A master Resilon cone size 35 taper 0.06 was placed and root canal was obturated by the same technique done in Groups 3 and 4.

The obturting material was cured for 30 seconds using visible light curing system after complete obturation of the root canals. To insure that the roots were perfectly obturated without any voids, postoperative periapical radiographs were taken for all obturated roots.

### 3.6. Root Reinforcement with Bonded Composite Resin and Glass Fiber Posts (Groups 4 and 6)

**Figure 2:**
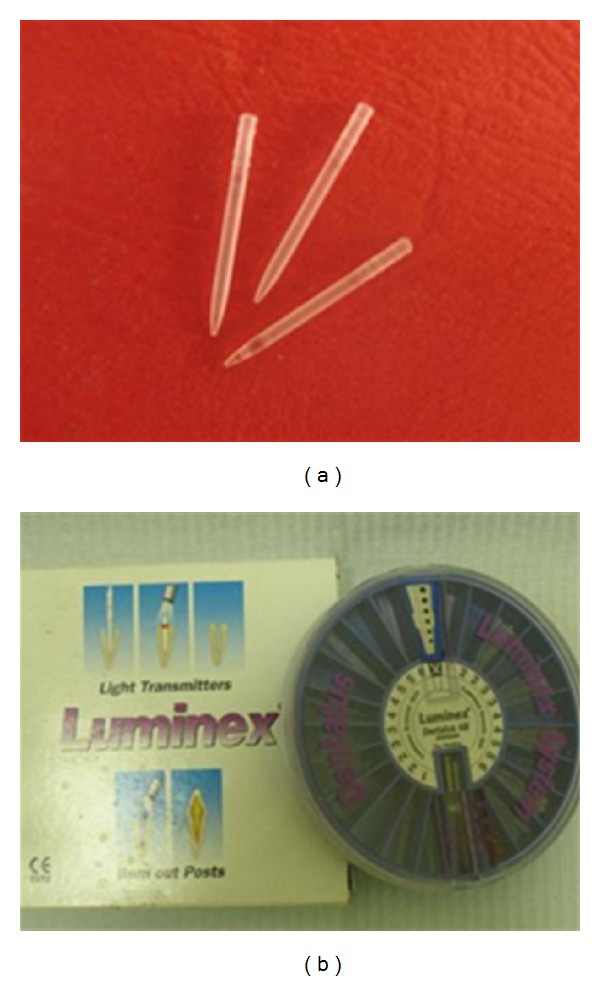
Plastic Transilluminating post (Luminex post system).

In Groups 4 and 6, the obturating materials were removed from the coronal 10 mm of each canal using Gates glidden drill sizes 3 and 4, without disturbing the apical 5 mm of the filling. Then the coronal 10 mm of each canal was weaken as previously mentioned in groups 2, 3, and 5. A radiograph was made to verify the removal of obturating materials and to assess whether the walls of the canal were clean and ready for bonding procedures. A light-transmitting plastic posts (Luminex post system Dentatus AB, Hagersten, Sweden) (Figure 2) size 3 were selected to facilitate curing of composite resin within the canal spaces. The root canal of each sample was etched for 20 seconds with 37% phosphoric acid etchant (Superetch, SDI Limited, Australia), rinsed with water using an irrigating syringe, and dried with paper points. A thin layer of dentin bonding agent (Excite, Ivoclar Vivadent) was applied using microbrush and light cured for 20 seconds. A light-cured composite (3 M Filtek SupremeXT) is syringed and condensed into the root canal using suitable prefitted hand pluggers.

**Figure 3:**
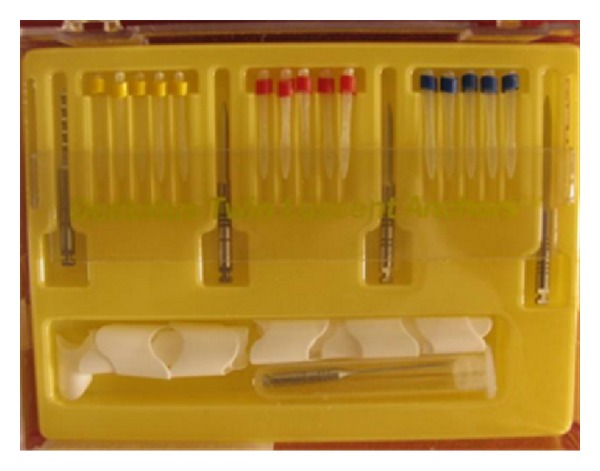
Luscent Anchor Posts.

The preselected plastic light-transmitting post was centered and fully seated through composite into the canal. The light-curing probe was placed directly over the Luminex post and the composite was light-cured multidirectionally for 2 minutes. The Luminex post was then removed and the space within composite was refined with Dentatus Helex Reamer size 4 (1.45 mm) to create 10 mm length post space within cured composite. Luscent Anchor post (Figure 3) (Dentatus USA Ltd) of the same size (size *S*, 1.45 mm) was selected and cut coronally to be 10 mm length and cleaned with a gauze soaked with alcohol. The adjusted Luscent Anchor post was then cemented with dual cured adhesive resin cement (Bistite II DC, Tokuyama Dental Corporation, Tokyo, Japan) and cured for 40-second following manufacturer's instructions.

## 4. Samples Fixation

All samples were stored for 1 week in an incubator (Memmert, Schwabach, Germany) at 100% humidity and 37°C to allow complete setting of the sealer. Aluminum rings of 30 mm in height and diameter were filled with self-cured acrylic resin (Sofa Dental, A KERR Company). Samples were embedded directly in acrylic leaving 3 mm of root structure unimpeded. A protractor was used to ensure that the long axis of the root sample was vertically aligned during the polymerization of the acrylic resin. To prevent overheating, samples were submerged in water for 6 minutes during resin polymerization.

## 5. Sample Holder

**Figure 4:**
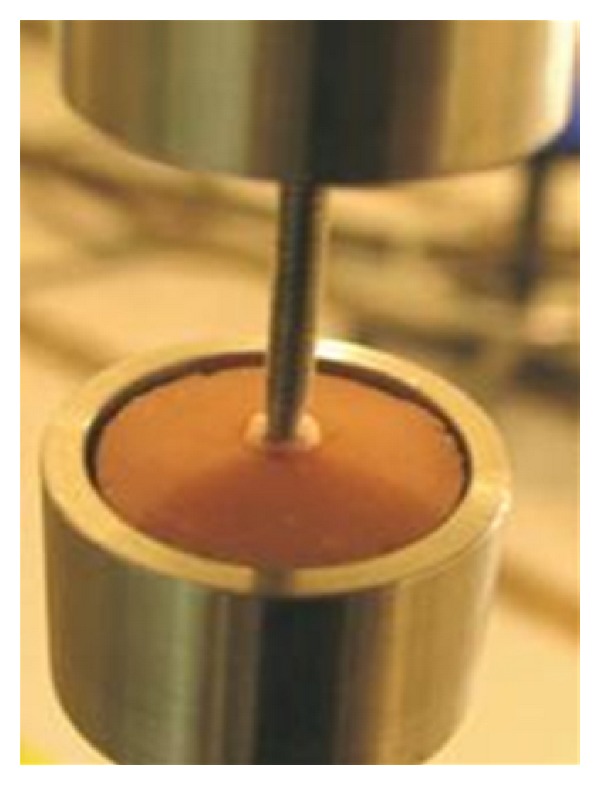
Spherical tip (*r* = 5 mm) was aligned with the center of the canal opening of each specimen.

A specially designed two sample holders were fabricated from round stainless steel rod with 80 cm length and 18 mm in diameter. One metallic rod was attached to the upper plate and the other rod was attached to the lower plate of a universal testing machine (DARTEC, USA Model Company). A metallic ball with 5 mm in diameter was attached to the upper metallic rod and a cylindrical metallic cup with the same dimension of the acrylic blocks was attached to the lower metallic rod (Figure 4). The holder was designed to (1) standardize measurements, (2) protect samples from tilting during measurements, and (3) provide good visibility of the sample throughout the measurements.

## 6. Fracture Resistance Measurement

All measurements were carried out at 21 ± 2°C using a computer-controlled universal testing machine (DARTEC, USA Model Company). The machine employs workshop 96, tool kit 96 and data manger software to analyze the measured data and plots the graphs. Each fixed sample was inserted into the lower part of the sample holder. The metallic ball (*r* = 5 mm) of the upper part of the holder was adjusted to be precisely on the opening of the root canal. A vertical load was applied for each specimen at a crosshead speed of 0.5 mm per minute until the root fractured. For this study fracture was defined as a point at which a sharp and instantaneous drop greater than 25% of the applied load will be observed. For most specimens, an audible crack also was observed. The test was terminated at this point and the force was recorded in Kilo-Newton. Averages and standard deviations were calculated and the data were analyzed by an analysis of variance (ANOVA) and multiple comparisons of means between all groups were performed with Tukey test using SPSS/PC version 12 (SPSS Inc., Chicago, IL, USA). Results with *P* < 0.05 were considered significant.

## 7. Results

**Figure 5:**
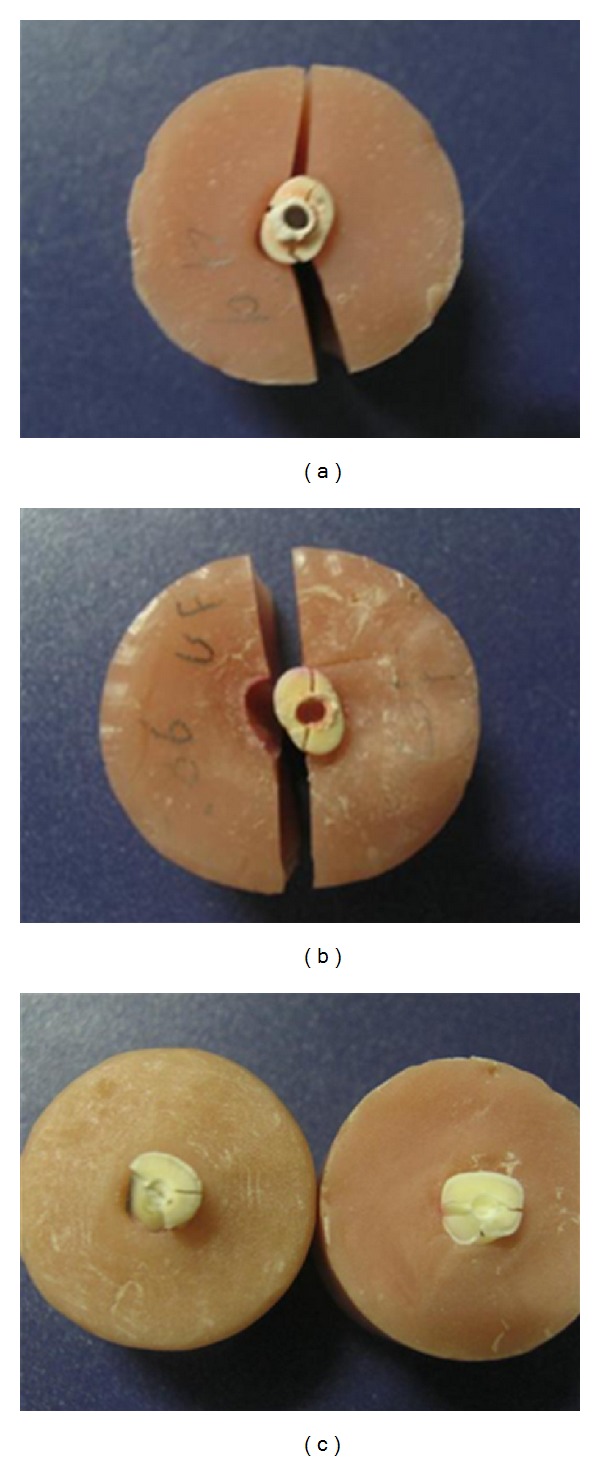
Patterns of root fracture.

 All roots showed horizontal and oblique fractures through their cervical area (Figure 5). Fracture strengths of all groups are shown in Table 2. The fractures extended more than 2 mm below the cervical margin of the roots in all groups except negative control that showed only cervical root fracture. The results of ANOVA test indicated a significant difference existed between the groups (*P* = 0.000). Post hoc analysis (Tukey's test) indicated the fracture resistance of roots that completely obturated with Resilon (Group 5) or restored with glass fiber posts and composite resin (Groups 4 and 6) was significantly superior to the unrestored positive controls (*P* = 0.0002) and the gutta-percha group (*P* = 0.0002). The differences between groups 1, 4, 5, and 6 were not significant (*P* ≥ 0.5785).

## 8. Discussion

This study was intended to evaluate and compare the effect of different restorative techniques on the strength of experimentally weakened endodontically treated roots. In the first technique (Group 3), the weakened root canals were directly obturated with gutta-percha and resin sealer. In the second technique, (Group 5) the canals were directly filled with bonded obturating material (Resilon system). In the third technique, the apical portion of canals was obturated with Resilon system (Group 6) or gutta-percha (Group 4) and then the remaining radicular portion was restored with a glass fiber post after rebuilding the lost coronal radicular dentin with light-cured composite resin using transilluminating plastic post (Luminex post system). The fracture resistance of the weakened roots restored with previously mentioned techniques was compared with the fracture resistance of unprepared roots (negative control, Group 1) and with unrestored prepared weakened roots (positive control, Group 2).

As this study was carried out on extracted single rooted teeth of different types, many uncontrollable variations that could affect the testing procedure were existed as was done in previous studies [35, 36]. To decrease some of these variations, all possible controllable factors were mostly standardized such root length (15 mm), apical foramen diameter (not more than size 15 file), cervical orifice diameter (2.5 mm), and cervical radicular dentin thickness (2.5 mm).

A standardized root canal preparation was performed using crown down rotary technique followed by a standardized overpreparation using post drills and specialized bur to weaken roots provided that the remaining radicular dentin thickness at the orifices was 1.5 mm. It was found that root canals with 1 mm of remaining buccal dentin walls were apparently more prone to fracture than that of 2 and 3 mm of dentin walls [37, 38]. Recapitulation and irrigation was done to re move any apical packing dentin. Final rinse with EDTA followed by distal water was performed to re move smear layer that could enhance bonding of sealer to the dentinal surface of the root [39]. Root weakening procedures for Groups 4 and 6 were performed after obturation to assure that all obturating materials were completely removed from the coronal portion of the canals. The apical 5 mm of obturating materials in Groups 4 and 6 was kept untouched as it was found that when a post is planned, at least 3–5 mm of obturating material must be remained apically to provide optimum apical seal [40–42].

 The effect of the periodontium was not reproduced through this study and all roots were embedded directly in acrylic blocks. Covering roots with silicon or wax before embedding in acrylic resin may cause root movement during loading which might not allow the study of actual behavior of used restorative technique. Furthermore, such periodontal membrane simulating materials have different elasticity than that of periodontium and thus are unrepresentative of the clinical status. Before mechanical testing, each root was embedded vertically in an acrylic resin block using a protractor leaving its coronal 2 mm was unimpeded. This design is more relevant clinically as it efficiently simulates the support given to healthy teeth by alveolar bone and results in less catastrophic stress build ups caused by unrealistic bending movements [43]. The teeth were submerged in water for 6 minutes during resin polymerization to prevent overheating.

 In several studies, tests for fracture strength were performed using the cyclic loading [44, 45] by applying the force in different directions in order to simulate the clinical conditions. However, in many studies, it has been reported that applying the force vertically to the long axis of the tooth transmits the force uniformly [46, 47]. In the present study, a single compressive load (with 0.5 mm/min cross head speed) was applied vertically as in many other studies that evaluated the fracture resistance of root filled teeth [48–50]. Higher cross-speed load application could cause impact instead of compression, which was the aim of the present study [50].

In the analysis of the current results, it was found that the fracture resistance of weakened unfilled roots was significantly much lower than that of unprepared roots. This result confirms the concept of the fracture resistance of endodontically treated roots is directly affected by remaining radicular dentin thickness at the cervical portion of the roots [51].

 Our results indicated that all restorative techniques (Groups 4–6) except gutta-percha and resin sealer technique (Group 3) could reinforce experimentally weakened roots. Gutta-percha and resin sealer could only improve fracture resistance of weakened roots but cannot reinforce them. The improvement of the fracture resistance of weakened roots in group 3 may be explained by following: the homogenous mass of gutta-percha and the resin sealer penetration within dentinal tubules could absorb the applied force and distribute stresses evenly along the radicular dentinal wall. In addition, the rounder canal preparation produced by rotary profile system may reduce area of stress concentration which may offset of increased dentin removed [52–55]. The inability of gutta-percha and resin sealer to reinforce weakened roots to the level of sound roots may be due lack of resin sealer bonding with radicular dentin and gutta-percha core [41].

 In spite of Resilon core has a very low modulus of elasticity in comparison with radicular dentin, [56] the using of Resilon system as a sole obturating material in the present study was able to reinforce weakened roots. Interestingly, the mean value of fracture load for these roots was higher than that of unprepared sound roots but without significant difference. The result is in agreement with Kazanday et al. [50] who studied the fracture resistance of roots using different canal filling systems and also with Schafer et al. [57] who studied the influence of resin-based adhesive root canal fillings on the resistance to fracture of endodontically treated roots. Our results is in disagreement with Ribeiro et al. [14], Carvalho et al. [58], Stuart et al. [59], Wilkinson et al. [60], Jainaen et al. [61], and Jainaen et al. [62]. The causes of disagreement with the other authors may be due to differences in the methodology of research such as roots dimensions, methods of canal preparation, obturation techniques, length of roots exposed to force, direction and method of force application, and finally the presence or absence of core material. Also our results are in disagreement with Williams et al. [56] who concluded that the cohesive strength and moduli of elasticity values for Resilon are too low to reinforce roots of endodontically treated teeth.

 The reinforcing effect of Resilon system as described in previous studies may be due to the chemical and mechanical bonding of Epiphany sealer to dentin and Resilon core material [50, 57, 63]. Also the large amount of Resilon material inside the wide canal and the bonded resin sealer could absorb and disperse shock energy [64]. Based on this result, the concept of monoblock described by many authors [34, 39] could be confirmed. So, when post and core are not indicated, Resilon system alone could be sufficient to reinforce the weakened roots.

In groups 4 and 6, reinforcement of the weakened roots can be obtained when they were restored with glass fiber posts after relining the flared canals with intermediate layer of resin composite cured with the aid of transilluminating post. The use of transilluminating post will create a post space within the cured composite that can be filled with either obturating materials or restored with any type of posts. In our study, glass fiber posts were selected to restore post space within composite resin as they have mechanical properties similar to dentin and have better bonding with composite resin [65, 66]. The weakened roots restored by this way showed the highest fracture resistance among all groups with no statistically significant differences with groups 1 (sound roots) and 5 (roots obturated with Resilon system). The monolithic structure of similar moduli of elasticity comprising dentin, resin composite, and glass fiber post may be the cause of the reinforcing effect of this restorative technique [67–70]. The results obtained in the current study are supported by many authors [71, 72] who suggested that adequate light polymerization of resin composite in the root canal with translucent posts would increase fracture resistance of endodontically treated roots. The current results are in disagreement with other studies which indicated that weakened roots restored with posts and different restorative materials did not able to achieve the fracture resistance recoded for unweakened roots [19, 73]. The causes of these disagreement may be due to differences in materials and post types, direction, and methods of force application.

 The present study was not able to determine the effect of glass fiber post on the fracture resistance of weakened roots that internally supported by light cured composite. The relining of wide canals with resin composite may be sufficient to increase the fracture resistance without using the glass fiber post. However, when post is indicated for core retention, relining of wide flared canals with bonded composite may be necessary before post cementation. There was no significant difference in the fracture resistance of weakened roots in Groups 4 and 6. This means that the type of obturating material in the apical portion of canals will not affect the fracture resistance of weakened roots restored coronally with glass fiber posts and composite but may affect apical leakage resistance.

 The current study evaluated only the maximum force that was needed to fracture roots and did not focus on the pattern of root fracture. This is because once root is fractured the tooth cannot be restored and will be extracted. A limitation of this study is the fact that it was performed in vitro and the results should be directly extrapolated to the clinical situations. Further studies should incorporate thermocycling.

## 9. Conclusions

Within the limits of the current study, it may be concluded that:

- weakened roots obturated with Resilon system alone or rebuilt with intermediate layer of composite resin bonded to radicular dentin and to glass fiber posts showed a significant increase in their fracture resistance.
- the transilluminating plastic post was a helpful method in rehabilitation of compromised root with light-cured composite.

## Figures and Tables

**Table 1:** Sample grouping.

Groups	No. of samples	Treatment
Group 1	10	No treatment
Group 2	10	Prepared weakened roots without obturation
Group 3	20	Prepared weakened roots obturated with gutta-percha and resin sealer
Group 4	20	Prepared weakened roots that apically obturated with gutta-percha and resin sealer and restored coronally with bonded composite resin and glass fiber post
Group 5	20	Prepared weakened roots obturated with resilon and epiphany resin sealer
Group 6	20	Prepared weakened roots that apically obturated with resilon and epiphany and restored coronally with bonded composite resin and glass fiber post

**Table 2:** Comparison of fracture strengths of all groups.

Groups	Treatment done	Mean ± S.D. (KN)*	Min (KN)*	Max (KN)*	ANOVA	
1	Unprepared roots	0.876 ± .327^a^	0.37	1.57	F	P
2	Prepared and unfilled weakened roots	0.193 ± .104^c^	0.06	.42	12.472	0.000
3	Roots filled with Gutta-percha only	0.618 ± .323^b^	0.24	1.21		
4	Roots apically filled with Gutta-percha and restored coronally with composite and post	0.970 ± .328^a^	0.29	1.39		
5	Roots filled with Resilon only	0.966 ± .775^a^	0.28	2.78		
6	Roots apically filled with Gutta-percha, and restored coronally with composite and post	1.014 ± .201^a^	0.69	1.35		

*Tukey post hoc test: means with the same superscript letter are not significantly different (*P* > 0.05).
